# Parental knowledge and communication with their adolescent on sexual and reproductive health issues in Nepal

**DOI:** 10.1371/journal.pone.0289116

**Published:** 2023-07-25

**Authors:** Devendra Raj Singh, Shreesha Shrestha, Kshitij Karki, Dev Ram Sunuwar, Dan Bahadur Khadka, Dikshya Maharjan, Lalita Kumari Sah, Bibha Simkhada, Rajeeb Kumar Sah

**Affiliations:** 1 Department of Public Health, Central Institute of Science and Technology (CIST) College, Pokhara University, Kathmandu, Nepal; 2 Department of Public Health, Asian College for Advance Studies, Purbanchal University, Satdobato, Lalitpur, Nepal; 3 School of Human and Health Sciences, University of Huddersfield, Huddersfield, United Kingdom; 4 School of Health and Life Sciences, Teesside University, Middlesbrough, United Kingdom; 5 Department of Nutritional Sciences, School of Public Health, University of Michigan, Ann Arbor, Michigan, United States of America; 6 Research and Innovation Section, Southeast Asia Development Action Network, Lalitpur, Nepal; 7 Ecole des hautes études en santé publique (EHESP School of Public Health), Rennes Cedex, France; 8 School of Nursing and Healthcare Leadership, University of Bradford, Bradford, United Kindom; University of Gondar, ETHIOPIA

## Abstract

**Background:**

Parental knowledge about sexual and reproductive health issues and adequate communication with their adolescent on these issues are crucial in promoting adolescent sexual and reproductive health. Although there are evidence on adolescent perceptions of their sexual health issues, research on parental perspectives of adolescent sexual health and parent-adolescent communication about sexual health issues in Nepal remains unexplored. Therefore, this study aimed to assess parental knowledge and communication practice about sexual and reproductive health with their adolescent children in Lalitpur Metropolitan City of Nepal.

**Methods:**

A community-based cross-sectional study was conducted between January and March 2019 among randomly selected 308 parents of adolescents (aged 10–19 years) residing in Lalitpur Metropolitan City of Nepal. Face-to-face interviews using structured questionnaires were conducted to collect the data. The collected data were entered into EpiData software v3.1, and data analysis was performed using IBM SPSS Statistics for Windows Version 21.0 (IBM Corp. Armonk, NY, USA). The statistical significance was considered at a p-value <0.05 and a 95% confidence interval (CI).

**Results:**

Of 308 parents, one-third of parents were found to have correct knowledge about safe abortion, menstrual hygiene and management, modern contraceptives, prevention of sexually transmitted infections, wet dreams among male adolescents, abstaining from sexual intercourse during the fertile period, and the possibility of a male adolescent to impregnate a girl. In addition, only 40.9% of parents were found to have communicated with their adolescent children about sexual and reproductive health issues. Parents who have knowledge about puberty (aOR = 2.2, 95% CI: 1.2–3.9), belong to *Bharamin/Chhetri* ethnic group (aOR = 1.2, 95% CI: 1.1–2.2), self-employed (aOR: 2.4, 95% CI: 1.3–4.0), having two or more adolescent children (aOR = 2.0, 95% CI: 1.1–3.6), and whose adolescent children were staying in school hostel (aOR = 1.7, 95% CI:1.0–3.0) were more likely to have parental communication about sexual and reproductive health with their adolescent children.

**Conclusions:**

Most parents do not communicate with their adolescent children on sexual health topics, although they feel sexual health education is essential to adolescents. The majority of parents were found inadequately aware of adolescent sexual health issues. It is crucial to have contextual interventions that would encourage parent-adolescent communication on sexual health matters in an integrated way to promote adolescent sexual and reproductive health.

## Background

Adolescent population aged 10−19 years is considered as one of the most vulnerable groups who encounter a range of sexual and reproductive health (SRH) issues [1, 2]. Their health issues have been prioritised globally to meet the Sustainable Development Goals (SDGs) target 3.7 [3]. Adolescents are more likely to initiate unsafe sexual activities that may put them at higher risk of experiencing unwanted pregnancy, unsafe abortion, and sexually transmitted infections, including HIV/AIDS [1, 4, 5]. Adolescents, particularly in low- and middle-income countries (LMICs), have inadequate sexual health knowledge and are exposed to misinformation on sexual health topics which have exacerbated their SRH issues [6, 7]. While school is considered as a key source for health information in most countries, parental communication with their adolescents is essential to transmitting knowledge, values, beliefs, social norms, expectations, and skills regarding SRH [8]. Hence, parental knowledge about adolescent’s SRH issues is imperative in imparting correct information to their children [9]. Moreover, cultural values and social norms also play a pivotal role in shaping the pattern and contents of discussions between parents and their adolescent children [8]. Positive communication between parents and their adolescents can help them to make informed decisions about their SRH issues [10]. However, there is lack of adequate evidence on parental knowledge and their communication with adolescents on sexual health issues.

Nepal, one of the resource-poor countries in South Asia, has approximately six million adolescent population, which accounts for 24% of the total population of Nepal [11]. Despite the progress in implementing a range of adolescent-friendly sexual health policies and interventions in Nepal, adolescents still lack access to adequate SRH information and services [12, 13]. The most recent nationally representative survey of Nepal showed a 17% prevalence of teenage pregnancy, a 32% unmet need for child spacing which is highest among married women aged 15–19, less than half (40%) of females aged 15−19 knew about legal provisions for safe abortion, 20% of teenage female have comprehensive knowledge of HIV/AIDS and 42% of married female aged 15−19 were already a mother [14]. In Nepalese culture, adolescent children usually stay with their parents, and they are expected to take permission from their parents for most of their decisions, including decision-making towards SRH issues. However, social stigma and deep-rooted cultural values restrict adolescent-parent communication and open conversations about SRH, even with close family members [12, 13, 15]. Adolescents in Nepal mainly discuss their SRH issues with their peers in the community and school due to the unfavourable familial environment to discuss such matters with their parents [12, 16]. Poor SRH knowledge among adolescents has posed greater risks of adverse sexual health consequences [16]. There is limited evidence from the Nepalese context to understand the level of parental knowledge about adolescent sexual health, which is necessary for parent-adolescent communication to support their adolescent children towards decision-making in SRH matters. Therefore, this study aimed to assess parental knowledge and communication practice about SRH with their adolescent children in Lalitpur Metropolitan City of Nepal.

## Materials and methods

### Study setting and design

A community-based cross-sectional study was conducted from January to March 2019 among parents of adolescent children aged 10−19 years residing in Lalitpur Metropolitan City of Nepal. It is one of the largest cities in Nepal, with a population of 284,942, an area of 15.43 square Kilometers, and 39 local administrative municipal wards [17]. This city is an integral part of Nepal’s capital region, called the Greater Kathmandu Valley, and is home to diverse population groups from different socio-cultural and ethnic backgrounds [17]. It was purposively selected as the study site due to its more comprehensive and diverse population composition, which can better represent the Nepalese urban population.

### Sample size determination and sampling procedure

The sample size was calculated using the single population proportion formula:

*n* = z^2^pq/e^2^ [18]

Where:

n = required sample size;

Z = Z-value associated with the desired level of confidence (i.e. 95% confidence level, Z = 1.96);

p = estimated proportion of the population with the characteristic of interest, i.e. 23% proportion (p) for parental communication with the adolescent child on SRH issues from the Indian context was considered [19]. Due to the lack of a similar study in Nepal, we took this estimate from the study conducted in India, Nepal’s neighbouring country with a similar socio-cultural context.

q = 1—p (the complement of the proportion with the characteristic of interest);

e = is the desired margin of error, i.e. 5% margin of error was considered.

Assuming a 15% non-response rate, the total calculated sample was 314. A systematic random sampling method was used to select the study participants [20]. First, all 29 local administrative units (locally called wards) of Lalitpur Metropolitan City were considered study area for this study. Second, considering diversity in representativeness, ten catchment locations in the two selected wards were randomly selected for the study sites. Since each of the wards has a large and diverse number of households, we use systematic random sampling techniques to select an equal number (30 households) of households from each catchment locations to meet the sample size of the study. We started by selecting a random household from the first catchment location chosen and then set every sampling interval (K^th^) household until the desired number was met within the catchment locations. The sampling interval (K^th^ value) was calculated by dividing the total number of households in the sampling frame by the calculated sample size of the study. The total number of households in the two selected wards was 6265. The calculated sampling interval was 20; therefore, K = 20^th^ was considered for selecting the households. Subsequently, either mother or father of the adolescent children of the selected households was interviewed. The parents (father or mother) of the adolescent children residing in the Lalitpur Metropolitan City of Nepal, who was available for an interview during enumerator visits to their home and who can speak Nepalese language, were included in the study. If both mother and father were willing to participate in the study, only one participant was selected through the lottery method. Therefore, the number of participants is equal to the number of households. Parents with serious health problems, including physical or psychological difficulties, or those absent on the data collection days were excluded from the study.

### Data collection and study variables

Data were collected through face-to-face structured interviews at the participant’s residence in the Nepalese language. The public health undergraduate students who were in their final year and were fluent in the local language worked as enumerators. The enumerators were provided three days of training on the study tools, data collection procedures, sample selection, potential ethical issues, and data handling procedures. As a result, all enumerators were familiar with the objectives and ethical aspects of the study. In addition, the field supervisor cross-checked the collected data daily on the study sites, and any missing data were re-inquired. The study tools were first developed in English and then translated into Nepali language. The back translation of the tools from Nepali to English language were done to ensure the original meaning of the tool after translation is consistent. Further, Nepalese versions of translated tools were pretested in adjoining municipalities among similar populations, and necessary corrections were made to ensure the consistency and appropriateness of the questionnaires.

The tools of this study were developed based on the previously used tools in similar studies conducted in the countries such as India and the Lao People’s Democratic Republic [9, 21]. Our study tools contain three different sections. The first section included the collection of demographic information such as participant’s age, sex, ethnicity, religion, type of family, education status, employment status, number of adolescent children, sex of adolescent children, and living arrangement of adolescent children. The second section included questions related to parents’ (participants) knowledge about adolescent SRH, including their knowledge about sexually transmitted infections (STIs). The 34 statements in the questionnaire were used under four broader headings: i) Knowledge about physical and emotional changes during the adolescent period, ii) Knowledge about menstruation, iii) Knowledge about pregnancy prevention, contraceptives, and safe abortion, and iv) Knowledge about sexually transmitted diseases. The third section included ten statements to assess parents’ (participants) perceptions of providing sexual health education to adolescent children. The responses to the statements were recorded into dichotomous answers, i.e. agree and disagree. The third section in the tool also included questions related to parental communication about SRH with their adolescent children, perceived barriers among parents regarding sexual health communication with their adolescent children, and their preferred preference of media for disseminating sexual health education to adolescents.

### Statistical analysis

The collected data were entered into EpiData software v3.1, and data analyses were performed using IBM SPSS Statistics for Windows Version 21.0 (IBM Corp. Armonk, NY, USA). The descriptive results are presented as mean, standard deviation, frequency, and percentage. To assess the association of outcome and covariates, binary logistic regression models were conducted separately for the two dichotomised outcomes: presence and absence of parental communication about SRH with their adolescent children. The independent variables significant in the unadjusted model were adjusted in the adjusted model. The statistical significance was considered at a p-value <0.05 and a 95% confidence interval (CI).

### Ethical aspects

The ethical approval of this study was obtained from Nepal Health Research Council (Ref. no: 813/2018). The Lalitpur metropolitan office also provided formal approval to conduct this study. The written consent from each participant was obtained before conducting face-to-face interviews with the participant. All participants were informed about their voluntary participation, their right to refuse or withdraw from the study at any point, and maintaining the confidentiality of their identity.

## Results

### Demographic characteristics of parents

A total of 308 parents (98.0%) of adolescent children with a mean age of 43.5 (± 5.5 SD) years were included in the analysis (Table 1). A total of 6 (1.91%) participants refused to provide complete information and were therefore excluded from the final analysis. Most participants were female (59.7%), from *Newar* ethnic group (68.5%), and followed *Hindu* religion (80.5%). About two-thirds (67.2%) lived in a joint family, had at least one adolescent child (68.5%), and adolescent children lived with their parents (64.6%). Among the total participants, 6.2% of participants had never attended school, 40.3% were self-employed, 41.9% had service-related jobs (private or public services), and 19.9% were homemakers. Three-quarters of the total (75.0%) parents had at least one male adolescent child (Table 1).

**Table 1:** Demographic characteristics of participants.

Variable		Number	Percentage
**Age (years)**	Mean ± SD (43.5 ± 5.5)		
**Sex**	Female	184	59.7
	Male	124	40.3
**Religion**	Hindu	248	80.5
	Non-Hindu	60	19.5
**Ethnicity**	Newar	211	68.5
	Brahmin/Chhetri	65	21.1
	Janjati, Dalits and minorities	32	10.4
**Education status**	Never attended school	19	6.2
	Basic education (1–8 grade)	77	25.0
	Secondary and above	212	68.8
**Employment status**	Services (Private and Government)	129	41.9
	Self-employed	124	40.3
	House maker and others	55	17.9
**Type of family**	Nuclear	207	67.2
	Joint and extended	101	32.8
**Parents having male adolescent children**	Yes	231	75.0
	No	77	25.0
**Parent having female adolescent children**	Yes	223	72.4
	No	85	27.6
**Number of adolescent children (boys and girls)**	One	211	68.5
	Two and more	97	31.5
**Living arrangement of adolescent children**	With parents	199	64.6
	Hostels/Not with parents	109	35.4

### Parent’s knowledge about adolescent sexual and reproductive health

More than two-thirds of the participants correctly responded about the typical start age for puberty (68.5%), development of sex organs during the puberty period (95.5%), stimulation for having erections and ejaculating (releasing sperm) in males (70.8%), Adolescents have a feeling of sexual arousal is normal (70.1%), attraction towards opposite sex is normal during puberty (70.4%) and changes in the skin of adolescents (70.1%) (Table 2). However, less than two-thirds responded correctly about the possibility of a male adolescent impregnating a girl (61.7%) and wet dreams as common during adolescence (60.1%). Comparatively, mother of adolescent children was found to have better knowledge about normal mensuration bleeding duration (90.2%), absorbent used during menstruation (84.2%), menstruation happens in girls once every 4–5 weeks (93%), and sanitary hygiene materials used during menstruation (64.1%). Similarly, higher percentages of father of adolescent children knew about emergency contraceptive methods (87.1%), modern contraceptive methods (65.3%), and using a condom for every act of sexual intercourse to prevent pregnancy (71.0%). However, less than half of the fathers and mothers have knowledge about safe abortion provisions and abstaining during *fertile windows*’ in the menstrual cycle to prevent pregnancy. Likewise, the majority of parents (97.4%) responded that sexually transmitted diseases are transmitted by sexual intercourse with an infected person and that having sex with sex workers is a risk for STIs (89.9%). However, only 55.5% and 62.0% of parents responded that contaminated blood transfusion and sharing of a sharp object could transmit STIs (Table 2).

**Table 2:** Parental knowledge about adolescent sexual and reproductive health (statements).

	Correct responses		
	N = 308	N = 124	N = 184
Statements	Overall n (%)	Father n (%)	Mother n (%)
**Knowledge about physical and emotional changes during the adolescent period**			
Puberty typically starts at 10–14 years for girls and 12–16 years for boys	211 (68.5)	89 (71.8)	122(66.3)
Development of sex organs during puberty period, such as development of breasts and hips in girls, and enlargement of the testicles and penis enlargement in boys	294 (95.5)	116 (93.5)	178 (96.7)
Adolescents have the development of pubic and underarm hair in both sex	227 (73.7)	93 (75.0)	134 (72.8)
The hormone testosterone is produced, which stimulates for having erections and ejaculating (releasing sperm) in male	218 (70.8)	88 (71.0)	130 (70.7)
Adolescents have a feeling of sexual arousal is normal	216 (70.1)	84(67.7)	132 (71.7)
Attraction toward the opposite sex is normal during puberty	230 (74.7)	97 (78.2)	133 (72.3)
It is possible for a male adolescent to impregnate the girl	190(61.7)	77 (62.1)	113 (61.4)
Wet dreams (nocturnal emissions) are common during the adolescent period.	185 (60.1)	80 (64.5)	105 (57.1)
Glands in the skin on the face, shoulders, and back start to become more active during puberty, producing more oil.	216 (70.1)	84 (67.7)	132 (71.7)
**Knowledge about menstruation**			
Knowledge about the start of average age at which girls commence mensuration	221(71.8)	91(73.4)	130(70.7)
Knowledge about normal mensuration bleeding duration	276(89.6)	110(88.7)	166(90.2)
Knowledge Absorbent that should be ideally used during menstruation	257(83.4)	102(82.3)	155(84.2)
Girl can go to school during menstruation	252(81.8)	101(81.5)	151(82.1)
Knew that it is foul-smelling during menstruation	256(83.1)	104(83.9)	152(82.6)
Knew pain during menstruation is not mean someone is sick	237(76.9)	103(83.1)	134(72.8)
Menstruation happens in girls once every 4–5 weeks	264(85.7)	92(74.2)	172(93.5)
Washing genitalia twice or more times per day during menstruation	151(49.0)	51(41.1)	100(54.3)
Hygiene Sanitary materials used during menstruation	166(53.9)	48(38.7)	118(64.1)
**Knowledge about pregnancy prevention, contraceptives, and safe abortion**			
Knowledge about modern contraceptive methods	200(64.9)	81(65.3)	119(64.7)
Knowledge about emergency contraceptive methods	248(80.5)	108(87.1)	140(76.9)
Knowledge about safe abortion provision	116(37.7)	39(31.5)	77(41.8)
Using a condom for every act of sexual intercourse prevents pregnancy	203(65.9)	88(71.0)	115(62.5)
Abstaining from sexual intercourse during the fertile window in the menstrual cycle prevents pregnancy	115(37.3)	49(39.5)	66(35.9)
**Knowledge about the sexually transmitted disease**			
Abstaining from sex completely prevents STIs	252(81.8)	104(83.9)	148(80.4)
Using condom for every act of sexual intercourse prevent STIs	203(65.9)	88(71.0)	115(62.5)
Sexual intercourse with an infected person can transmit STIs	300(97.4)	122(98.4)	178(96.7)
Sharing of a sharp object can transmit STIs	191(62.0)	76(61.3)	115(62.5)
Contaminated blood transfusion can transmit STIs	171(55.5)	70(56.5)	101(54.9)
Having sex with sex workers is a risk for STIs	277(89.9)	116(93.5)	161(87.5)
Sharing of a toilet can transmit STIs	292(94.8)	114(91.9)	178 (96.7)
Kissing can transmit STIs	263(85.4)	109(87.9)	154(83.7)

### Parent’s perception towards providing sex education to adolescent children

Majority of parents agreed that sex education is essential for adolescents (98.4%), wish to provide sex education to adolescent children (86.3%), and both mother and father are responsible for providing sex education to their adolescent children (91.1%). However, only 28.2% of parents agreed that adolescents should only be provided with sexual health education if their adolescent children ask for it. Less than half (46.0%) of the parents agreed that 10–14 years of age is appropriate for providing sexual health education. Similarly, 81% of mothers and 79.9% of fathers of adolescents children agreed that parents should be included in sexual health education programs. More than two-thirds (76.6%) of parents agreed that school is the right place to discuss sex education for children, whereas only 54% agreed that parents are an appropriate source of sex education for children (Table 3). Parents preferred means for disseminating sex education to adolescents was course book (87.5%) followed by television (78.5%), radio (37.3%), internet (26.4%) and newspaper (16.8%) (Table 3).

**Table 3:** Parents’ perception towards providing sex education to adolescent children.

Statements	Agree with the statements		
	Father	Mother	Total
	n (%)	n (%)	n (%)
Sex education is essential for adolescents	121(97.6)	182(98.9)	303(98.4)
10–14 years of age is appropriate for providing sex education	57(46.0)	91(49.5)	148(48.1)
Adolescents should only provide sex education only if their children seek such education	35(28.2)	60(32.6)	95(30.8)
Wish to provide sex education to adolescent children	107(86.3)	160(87)	267(86.7)
Parents are an appropriate source of sexual health education for children	67(54.0)	87(47.3)	154(50.0)
School is the right place to discuss sex education for children	95(76.6)	136(73.9)	231(75.0)
Mothers should teach about sexual health to their girl child	80(64.5)	122(66.3)	202(65.6)
Fathers should teach about sexual health to their male child	77(62.1)	126(68.5)	203(65.9)
Both mother and father are responsible for providing sex education to their adolescent children	113(91.1)	178(96.7)	291(94.5)
Should parents be included in the sexual health education program	97(78.2)	149(81.0)	246(79.9)
**Preference of media for disseminating sex education to adolescents (multiple responses)**			**Percentage**
Television			78.5
Radio			37.3
Internet sources			26.4
Course book			87.5
Newspaper and magazine			16.8

### Parental communication about sexual and reproductive health with their adolescent children

**Fig 1:**
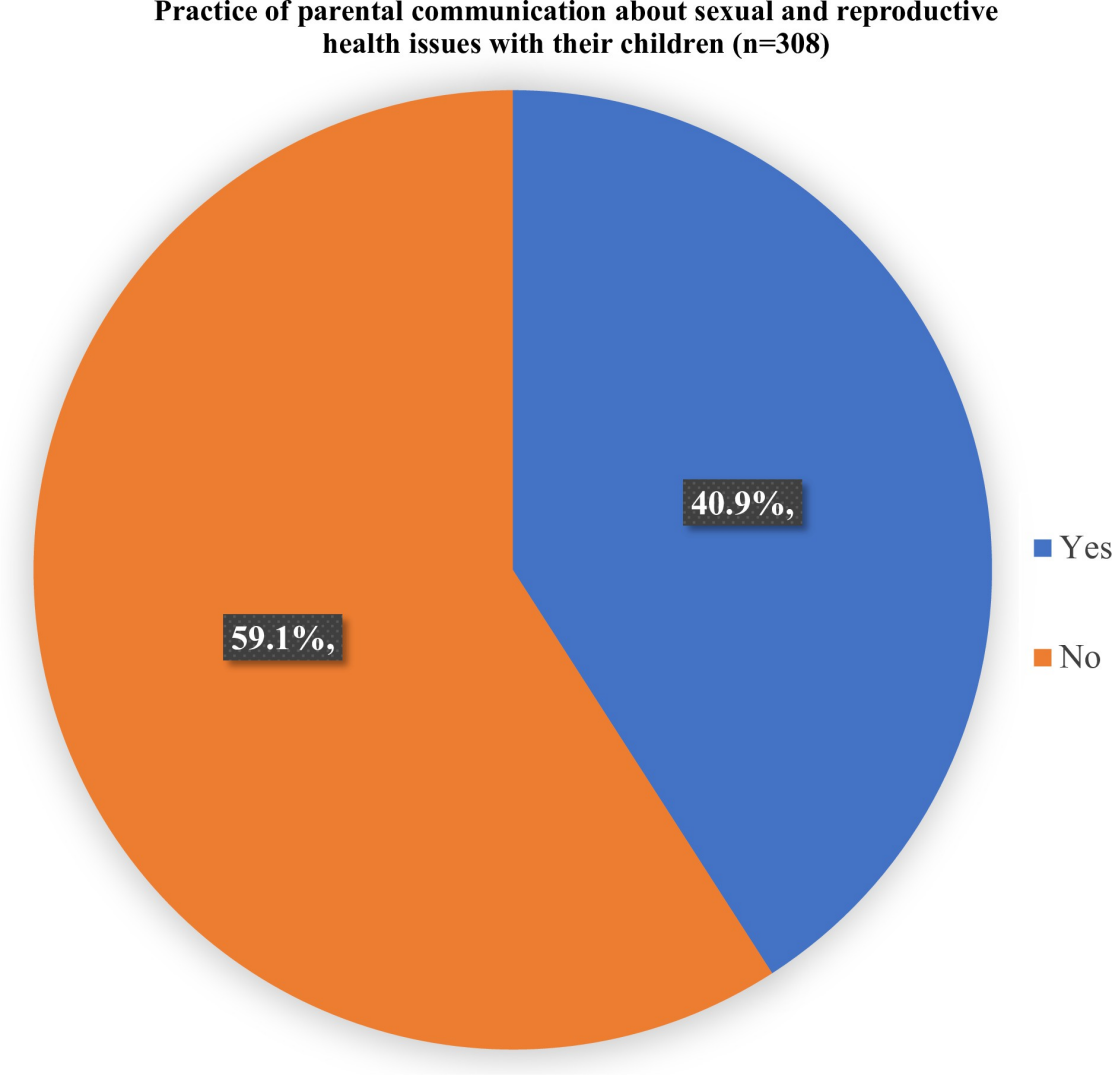
Parental communication about sexual and reproductive health with their adolescent children.

Less than half (40.9%) of the parents were found to have practised parent-adolescent communication about SRH issues (Fig 1). Among parents who communicated with their children about SRH issues, only 62.7% discussed menstrual hygiene practices, 54.8% discussed physical and emotional changes in adolescents, 34.9% discussed safe sex, and 37.3% discussed the prevention of STIs. Similarly, less than one-third of the parents were found to have discussed safe abortion practices (20.6%), different types of contraceptive use |(29.4%), appropriate age at pregnancy and childbirth (28.6%), and availability of adolescent-friendly sexual and reproductive services (17.5%) (Table 4).

**Table 4:** Parental communication about different types of sexual and reproductive health issues with their adolescent children.

Type of sexual and reproductive health issues discussion with their adolescent children (n = 116)	
Discuss about physical and emotion changes in adolescents	69(54.8)
Discuss about safe abortion practices	26(20.6)
Discuss about hygienic practice of menstrual hygiene	79(62.7)
Discuss about safe sex	44(34.9)
Discuss about prevention from STIs	47(37.3)
Discuss about different types of contraceptives uses	37(29.4)
Discuss about appropriate age at pregnancy and child birth	36(28.6)
Discuss about availability of adolescent-friendly sexual and reproductive services	22(17.5)

### Perceived barriers for parents in sexual health communication with adolescent children

**Fig 2:**
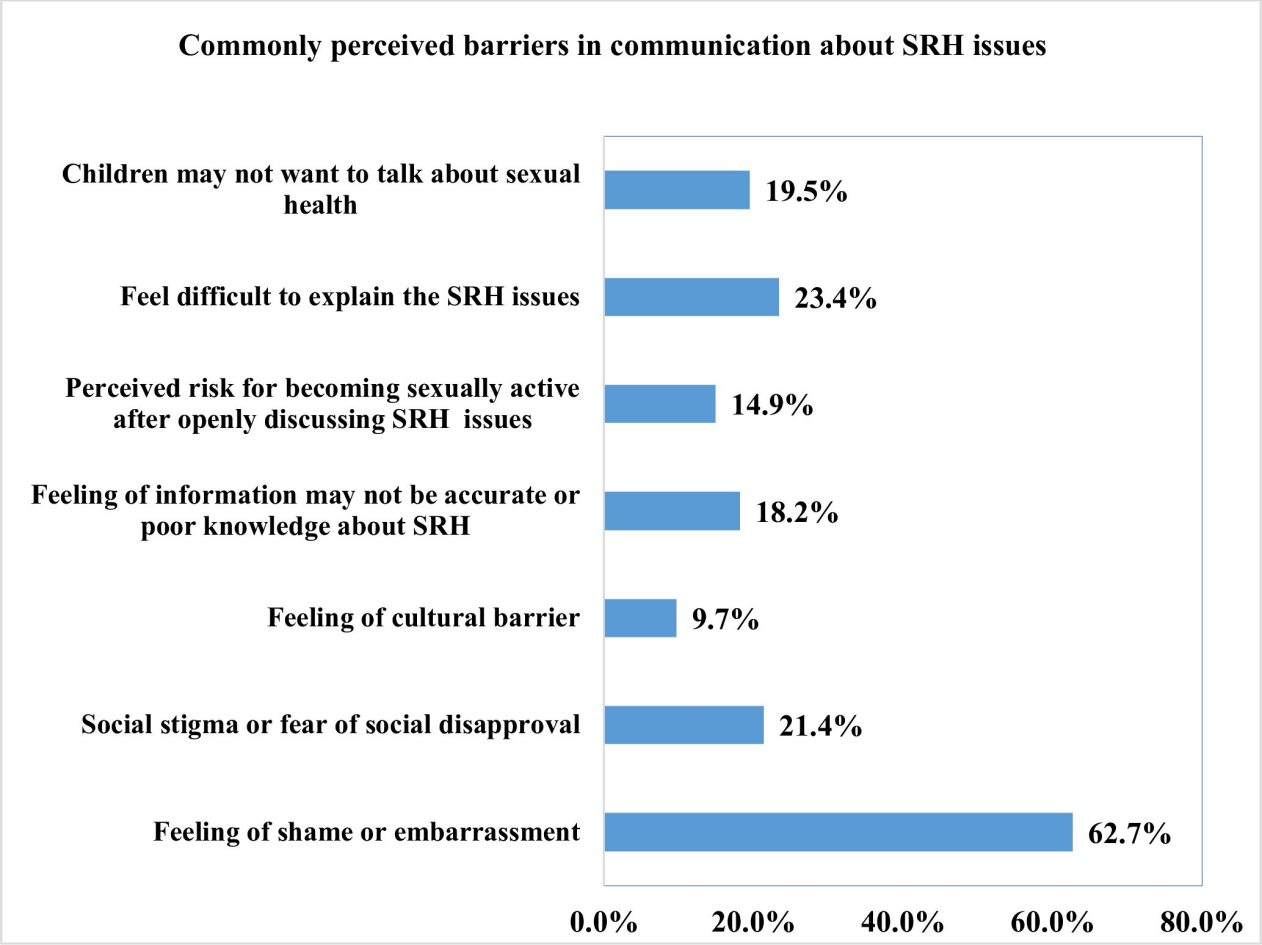
Perceived barriers for parents in communicating about sexual health issues with adolescents children.

More than half (62.7%) of parents mentioned that feelings of shame or embarrassment as a key barrier in communication about sexual health with their adolescent children. Similarly, feelings of difficulty explaining SRH issues (23.4%), social stigma or fear of social disapproval (21.4%), children may not want to talk about sexual health (19.5%), feeling of information may not be accurate or poor knowledge about SRH (18.2%), perceived risk for becoming sexually active after openly discussing SRH issues (14.9%), and feeling of cultural barrier (9.7%) were other perceived barriers for parent-adolescent sexual communication (Fig 2).

### Factors associated with parental communication about sexual and reproductive health with their adolescent children

In unadjusted bivariate logistic regression analyses, participants’ sex, ethnicity, employment status, family type, number of adolescent children, adolescents living arrangements, and parental knowledge about puberty were significantly associated with parental communication about SRH with their adolescent children (Table 5). In the multivariate logistic regression (Table 5), participants’ ethnicity, employment status, number of adolescent children, adolescents living arrangements, and parental knowledge about puberty variables remained statistically significant after adjusting for covariates. Compared to *Newar* ethnic group to *Brahmin/Chhetri* (aOR = 1.2, 95% CI = 1.1–2.2), adolescent children living in a school hostel or with their relatives (aOR = 1.7, 95% CI = 1.0–3.0) and have two or more adolescents’ children (aOR = 2.0, 95% CI = 1.1–3.6) were associated with higher odds of having parental communication about SRH with their adolescent children. Similarly, self-employed parents (aOR = 2.3, 95% CI = 1.3–4.0), and parents who have knowledge about puberty (aOR = 2.2, 95% CI = 1.2–3.9) were more likely to have parental communication about SRH with their adolescent children.

**Table 5:** Unadjusted and adjusted odds ratios using logistic regression for factors associated with parental communication about sexual and reproductive health with their adolescent children.

Variables	parental communication about sexual and reproductive health with their adolescent children		Bivariate analysis (unadjusted)	Multivariate analysis (adjusted)
	Yes	No	cOR (95% CI)	aOR (95% CI)
	n(%)	n(%)		
**Sex**				
Male	39(31.5)	85(68.5)	Ref	Ref
Female	87(47.3)	97(52.7)	1.9(1.2–3.1)*	1.5(0.8–2.8)
**Religion**				
Hindu	99(39.9)	149(60.1)	Ref	-
Non-Hindu	27(45.0)	33(55.0)	0.8(0.4–1.4)	-
**Ethnicity**				
Newar	81(38.4)	130(61.6)	Ref	Ref
Bhramin/Chhetri	26(40.0)	39(60.0)	0.9(0.5–1.6)*	1.2(1.1–2.2)*
Other castes	19(59.4)	13(40.6)	0.4(0.2–0.9)*	0.4(0.1–0.9)
**Education status**				
Never attended school	11(28.2)	28(71.8)	Ref	-
Basic education	21(26.9)	57(73.1)	1.0(0.3–3.2)	-
Secondary and above	94(49.2)	57(50.8)	0.5(0.2–1.4)	-
**Employment status**				
Public or private Services	68(52.7)	61(47.3)	Ref	Ref
Self–employed	35(28.2)	89(71.8)	2.8(1.6–4.7)*	2.3(1.3–4.0)*
Homemaker and others	23(41.8)	32(58.2)	1.5(0.8–2.9)	1.5(0.7–3.1)
**Family type**				
Nuclear	102(49.3)	105(50.7)	Ref	Ref
Joint	24(23.8)	77(76.2)	3.1(1.8–5.3)*	1.5(0.8–2.8)
**Number of adolescent children**				
One	98(464)	113(53.6)	Ref	Ref
Two and more	28(28.9)	69(71.1)	2.1(1.2–3.5)*	2.0(1.1–3.6)*
**Adolescent living arrangement**				
With parents	91(45.7)	108(54.3)	Ref	Ref
With Relatives or stay in a school hostel	35(32.1)	74(67.9)	1.7(1.0–2.9)*	1.7(1.0–3.0)*
**Knowledge about puberty**				
No	30(30.9)	67(69.1)	Ref	Ref
Yes	96(45.5)	115(54.5)	1.8(1.12–3.1)*	2.2(1.2–3.9)*
**Knowledge about the appropriate age for providing sex education**				
No	54(33.8)	106(66.2)	Ref	-
Yes	72(48.6)	76(51.4)	1.8(1.1–2.9)	-

*p < 0.05; Ref: reference category; cOR: Crude Odds Ratio, aOR: Adjusted Odds Ratio; CI Confidence Interval, ^a^Adjusted for variables significant in the unadjusted model.

## Discussion

Parental communication with adolescent children is crucial in enhancing SRH outcomes of adolescents. This study provides insight into Nepalese parents’ knowledge and communication about SRH with their adolescent children. This study revealed that less than two third of parents had adequate knowledge about legal abortion in Nepal, wet dreams among male adolescents, abstaining from sexual intercourse during the fertile period, and the possibility for a male adolescent to impregnate a girl. These results are supported by the study conducted in Iran, where despite the parent’s negative attitude toward teenage pregnancy among their daughter, they had a low level of knowledge about the health consequences of pregnancy at an early age [22]. Likewise, a similar study from Nepal also disclosed that adolescents felt unsupported by their parents and family in accessing reproductive health information and services [13]. Women’s poor knowledge about safe abortion services, cultural attitudes, religious beliefs, cultural norms, and stigma were key barriers to seeking SRH services and information among Nepalese women [23, 24]. This may be due to poor health literacy and lack of access to adequate health information among the adult population in resource-limited countries such as Nepal [25].

Moreover, this study showed that fathers were relatively less aware of mensturation hygiene and management issues compared to mothers. These results are parallel to the findings from the study conducted in India, which reflects that males had a vague and inadequate understanding of menstrual hygiene and management issues [26]. In Nepalese society, cultural taboos and stigma restrict males from talking openly about female sexual health issues could be the reason for limited awareness of menstrual management issues among fathers of female adolescents [15].

Despite the majority of parents in this study perceived that SRH education is essential to their adolescent children, nearly half of them perceived that parents are not an appropriate source for sexual health education for their children and that adolescents should be provided with sexual health education only on children’s demands. These findings resemble the results from a similar study conducted in the neighbouring country India [9]. The study in the Indian context showed that majority of parents perceived that adolescent children should receive sexual health information from other sources rather than parents, as parents feel it is wrong to have sexual health issues conversations with their children [9]. Likewise, our study revealed that less than half of the parents had communicated about sexual health topics with their adolescent children. These findings are comparable with a similar study conducted in the Kailali district of western Nepal. More than half adolescents had never communicated on SRH topics with their parents [12]. In addition, similar to the previous studies [9, 12, 13, 15], most parents in this study perceived difficulty in explaining SRH issues. Social stigma or fear of social disapproval and feelings of lack of sexual health information were seen as barriers to limited parental-adolescent communication on sexual health matters. These results seemingly indicate powerful gender and sexual norms rooted within the Nepalese society that prohibits open discussions with family members on SRH issues [13, 27, 28]. These deeply rooted socio-culture norms and gender differences are believed to control all aspects of socialisation in the family context, including the practice of parental and adolescent communication about sexual health issues [12, 13].

This study showed that parents from *Brahmin/Chhetri* ethnic group were more likely to communicate about sexual health matters with their adolescent children compared to the *Newar* ethnic group. These ethnic differences in communication about sexual health issues reflect the difference in the socio-cultural structure of Nepalese society based on caste and ethnicity. *Brahmin/Chheteri* are comparatively placed higher in Nepal’s caste/ethnicity hierarchy. Therefore, they are considered privileged groups with better access to education, socioeconomic circumstances, and life opportunities [29]. Moreover, the study disclosed that parents who have knowledge about puberty, are self-employed, have two or more adolescent children, and have adolescent children living in school hostels were found to be in a better position with higher chances of communicating with their children about sexual health issues. These results are supported by the evidence from Namibia, where parents found it difficult to provide sexual health education to their children because they do not have adequate sexual health knowledge and do not know how or when to start sexual health communication and what SRH issues to communicate to their adolescent children [30]. Likewise, self-employed parents might have adequate time to share with their children since most of the parents residing in an urban region in Nepal are more concerned with their careers and jobs, and they have less leisure time for their children due to their busy schedules [31]. Moreover, parents were concerned about their adolescent children being at a higher risk of engaging in unsafe sexual activities while they were away from home [32, 33], which might have compelled parents to communicate about sexual health with their children who usually live in the school hostel.

Nonetheless, the results from this study explicitly suggest that parents in Nepal are not providing their adolescents with the information that will support their adolescents to experience their sexual health positively. That is to say, parents are reluctant to engage in open conversations with their adolescent children on sexual health issues even though the majority of parents in our study realised that sexual health education is essential for adolescent sexual development.

## Limitations and strengths of the study

The results of this study need to be viewed based on the strength and limitations of this study. First, since this study is cross-sectional, the association between cause and effect between independent and dependent variables cannot be confirmed. Second, the study was conducted in the urban setting of Nepal, sothe results of this study may not be generalised to the rural context of the country. Third, since the information was collected through face-to-face interviews among parents of adolescent children using questionnaires may lead to social desirability bias. Despite the limitations, this is one of the first studies to generate evidence from parents of adolescent children regarding the knowledge and practice of parent-adolescent communication about sexual health issues. The results of this study can be used as evidence for developing interventions not only to educate adolescents but also to educate and encourage parents for their necessary engagement in the discussion of sexual health issues with their adolescent children that can help in the protection and promotion of adolescent sexual health in Nepal. Also, the study has set the background for conducting qualitative studies to get an in-depth understanding of the poor engagement of parents in communicating sexual health knowledge to their adolescents.

## Conclusion

The study concludes that most parents do not communicate on sexual health topics with their adolescent children, although they feel sexual health education is essential for their adolescent children. Most parents were found inadequately aware of adolescent sexual health issues. The feeling of shame or embarrassment, cultural barriers, social stigma, inadequate knowledge about sexual health, and lack of skills for communication about the SRH issues were seen as major barriers to preventing parental and adolescent communication about sexual health matters. This study recommends that parents require support to be able to meet the sexual health information needs of their adolescent children. Parent’s ethnicity, knowledge about puberty, number of adolescent children, adolescent children living arrangements, and parent’s employment types were associated with parent-adolescent communication about sexual health issues. Thus, the study results urge appropriately designed contextual intervention that can create awareness and motivate parents to improve their engagement in promoting the SRH of their adolescent children. The findings also warrant the development of culturally appropriate local policy that will guide concerned stakeholders at the community level with the involvement in the integrated way of adolescent sexual and reproductive health promotion in Nepal. Nevertheless, the adolescent should be able to get sexual health information from multiple sources, including parents, family, friends, school, and the community.

## Supporting information

S1 Data (SAV)

## List of abbreviations

AIDS: Acquired Immunodeficiency Syndrome
CI: Confidence Interval
HIV: Human Immunodeficiency Virus
LMICs: Low- and Middle-income Countries
SD: Standard Deviation
SDGs: Sustainable Development Goals
SRH: Sexual and Reproductive Health
WHO: World Health Organization
